# Emerging Detection Techniques for Large Vessel Occlusion Stroke: A Scoping Review

**DOI:** 10.3389/fneur.2021.780324

**Published:** 2022-01-06

**Authors:** Jennifer K. Nicholls, Jonathan Ince, Jatinder S. Minhas, Emma M. L. Chung

**Affiliations:** ^1^Department of Cardiovascular Sciences, University of Leicester, Leicester, United Kingdom; ^2^Department of Medical Physics, University Hospitals of Leicester, NHS Trust, Leicester, United Kingdom; ^3^NIHR Leicester Biomedical Research Centre, University of Leicester, Leicester, United Kingdom; ^4^School of Life Course Sciences, King's College London, London, United Kingdom

**Keywords:** large vessel occlusion, LVO, stroke, stroke scale, biomarkers, algorithms, machine learning, imaging

## Abstract

**Background:** Large vessel occlusion (LVO) is the obstruction of large, proximal cerebral arteries and can account for up to 46% of acute ischaemic stroke (AIS) when both the A2 and P2 segments are included (from the anterior and posterior cerebral arteries). It is of paramount importance that LVO is promptly recognised to provide timely and effective acute stroke management. This review aims to scope recent literature to identify new emerging detection techniques for LVO. As a good comparator throughout this review, the commonly used National Institutes of Health Stroke Scale (NIHSS), at a cut-off of ≥11, has been reported to have a sensitivity of 86% and a specificity of 60% for LVO.

**Methods:** Four electronic databases (Medline *via* OVID, CINAHL, Scopus, and Web of Science), and grey literature using OpenGrey, were systematically searched for published literature investigating developments in detection methods for LVO, reported from 2015 to 2021. The protocol for the search was published with the Open Science Framework (10.17605/OSF.IO/A98KN). Two independent researchers screened the titles, abstracts, and full texts of the articles, assessing their eligibility for inclusion.

**Results:** The search identified 5,082 articles, in which 2,265 articles were screened to assess their eligibility. Sixty-two studies remained following full-text screening. LVO detection techniques were categorised into 5 groups: stroke scales (*n* = 30), imaging and physiological methods (*n* = 15), algorithmic and machine learning approaches (*n* = 9), physical symptoms (*n* = 5), and biomarkers (*n* = 3).

**Conclusions:** This scoping review has explored literature on novel and advancements in pre-existing detection methods for LVO. The results of this review highlight LVO detection techniques, such as stroke scales and biomarkers, with good sensitivity and specificity performance, whilst also showing advancements to support existing LVO confirmatory methods, such as neuroimaging.

## Introduction

Large vessel occlusion (LVO) is the obstruction of large, proximal cerebral arteries and accounts for 24–46% of acute ischaemic stroke (AIS), when including both A2 and P2 segments of the anterior and posterior cerebral arteries (1). Due to the involvement of proximal vasculature, significant brain regions are often affected, resulting in large neurological deficits (2).

Over the last decade, LVO care has been extensively researched and advanced, with strategies to allow earlier diagnosis and improved occlusion management. Advancements in the use of mechanical thrombectomy (MT) have played a critical role in improving LVO care (3). MT is usually performed at a comprehensive stroke centre (CSC), a hospital with complex endovascular facilities. Patients brought directly to a CSC for MT follow the “mothership” paradigm. An alternative pathway to the mothership is the “drip and ship” pathway, known as the primary care pathway—here, patients are sent to the nearest stroke centre that provides intravenous thrombolysis (IVT) as early as possible. Normal standard of care for LVO patients is IVT prior to MT (4). However, some LVO patients may require further transportation to a CSC for MT, increasing the time between the onset of first symptoms and time to reperfusion (4). Consequently, to improve patient outcomes and to minimise the time to reperfusion, early detection and direct transportation to a CSC *via* the mothership pathway should be considered. This was highlighted in recent American Heart Association (AHA) Guidelines (2019), which also called for research in identifying effective pre-hospital procedures for triaging patients to the most appropriate centres, including operational bypass algorithms (5). Specialised ambulances, known as mobile stroke units, are now providing rapid evaluation in-field using built-in computed tomography (CT) scanners for LVO identification, which may help to decrease the number of unnecessary patient transfers to hospital (6).

Typically, the detection of stroke initially relies on clinical presentation and the use of stroke triaging scales, followed by confirmatory neuroimaging in hospital. Whilst it is generally agreed that computed tomography (CT) and magnetic resonance imaging (MRI) are the gold standard for confirmatory stroke imaging, the consensus on optimal detection and triaging pathways prior to this neuroimaging is less clear. This was also discussed in the recent AHA Guidelines (2019), which called for better pre-hospital stroke identification tools and found no clear evidence for one tool over another (5).

In addition to the use of established stroke prediction scales, recent literature has proposed improved scales and novel detection techniques, including biomarkers, new imaging modalities, clinical manifestations, and decision-focused algorithms combining these.

The selection of optimal detection and triaging methods is critical for supporting the recent advancements in LVO management. Careful selection of techniques with high sensitivity will ensure all LVO patients are correctly identified and triaged to a suitable treatment centre, but may also result in a significant number of false positives. Favouring a high specificity may identify all non-LVO patients, preventing them from being unnecessarily triaged for LVO investigation and care, hence reducing system burden. However, a high specificity may also increase the number of false negatives identified. Ideally, LVO detection methods would have both high sensitivity and specificity. Nevertheless, it is important to recognise that sensitivity and specificity exist in balance; an increased sensitivity often comes at the cost of a reduced specificity, and vice versa. Detection techniques with sensitivity and specificity values of <50% could suggest they are worse than chance. As a good comparator throughout this review, the commonly used National Institutes of Health Stroke Scale (NIHSS), at a cut-off of ≥11, has been reported to have a sensitivity of 86% and a specificity of 60% for LVO (7).

This scoping review aims to explore existing literature in LVO detection techniques to identify any advancements over the last 5 years. This review will then collate relevant records to identify gaps in the current literature for future work. These aims will be achieved through describing and categorising relevant records, evaluating the sensitivity and specificity of detection methods, and exploring advantages and disadvantages of different detection methods.

## Methods

This scoping review search was conducted using the preferred reporting items for systematic reviews and meta-analyses (PRISMA) extension for scoping reviews and the PRISMA scoping review (PRISMA-ScR) checklist (8). There is no expectation for a bias assessment on the scoping PRISMA-ScR, so no formal risk of bias assessment is included in this review. Prior to the search, the protocol was registered with the Open Science Framework (10.17605/OSF.IO/A98KN).

### Search Strategy and Eligibility Criteria

A systematic search was conducted on the 22nd March 2021 using a pre-agreed search strategy (see Supplementary Table S1). This strategy was developed with assistance from a Library Research Services Consultant, Library Research Services, University of Leicester, and used truncations to narrow or expand search terms. The search was conducted using four electronic databases: MEDLINE *via* OVID (1946-current), SCOPUS (1966-current), CINAHL (1961–present), and Web of Science Core Collection (1970–current). Grey literature was searched using the OpenGrey database.

Limits applied to the search included human participant studies and English language manuscripts. No geographical restrictions were applied. Only records published from January 2015 to the end of February 2021 were included.

Retrieved records were imported into Mendeley (version 1.19.8) and automated duplication removal was applied. Two reviewers (JKN and JI) independently screened the titles and abstracts of records to determine their suitability according to the pre-determined inclusion and exclusion criteria. Only manuscripts reporting primary research studies (any study design type) and conference proceedings were included to identify emerging LVO detection methods. The population identified was LVO stroke in clinical and non-clinical populations, in either clinical or non-clinical environments. Only records reporting novel techniques developed since 2015 for LVO detection that had not yet been validated or advancements in pre-existing detection techniques were kept. Records with no full-text available were excluded.

### Selection of Articles for Inclusion

After initial screening, suitable records underwent full text review to determine their relevance to the research question. Studies were assessed for their eligibility based on their objectives, study design, key findings, and conclusions.

### Data Charting

A standardised data charting form (see Supplementary Table S2) was used to extract key findings from the final retrieved records. Any uncertainties raised during data charting were discussed between investigators. Data charting allowed collated information to be more readily compared for discussion.

### Collating, Summarising and Reporting Findings

A scoping review was performed due to the broad types of data collected; this meant there was no a-priori plan for data meta-analysis. Included records were then categorised based on their investigated detection type, including stroke scales, imaging and physiological methods, algorithmic and machine learning approaches, physical symptoms, and biomarkers.

## Results

The search identified 5,082 records. Duplicates (*n* = 2,275) were automatically removed using Mendeley, resulting in 2,807 retrieved records. Initial title and abstract screening resulted in 2,665 records being excluded due to being outside of the context of the research question.

**Figure 1:**
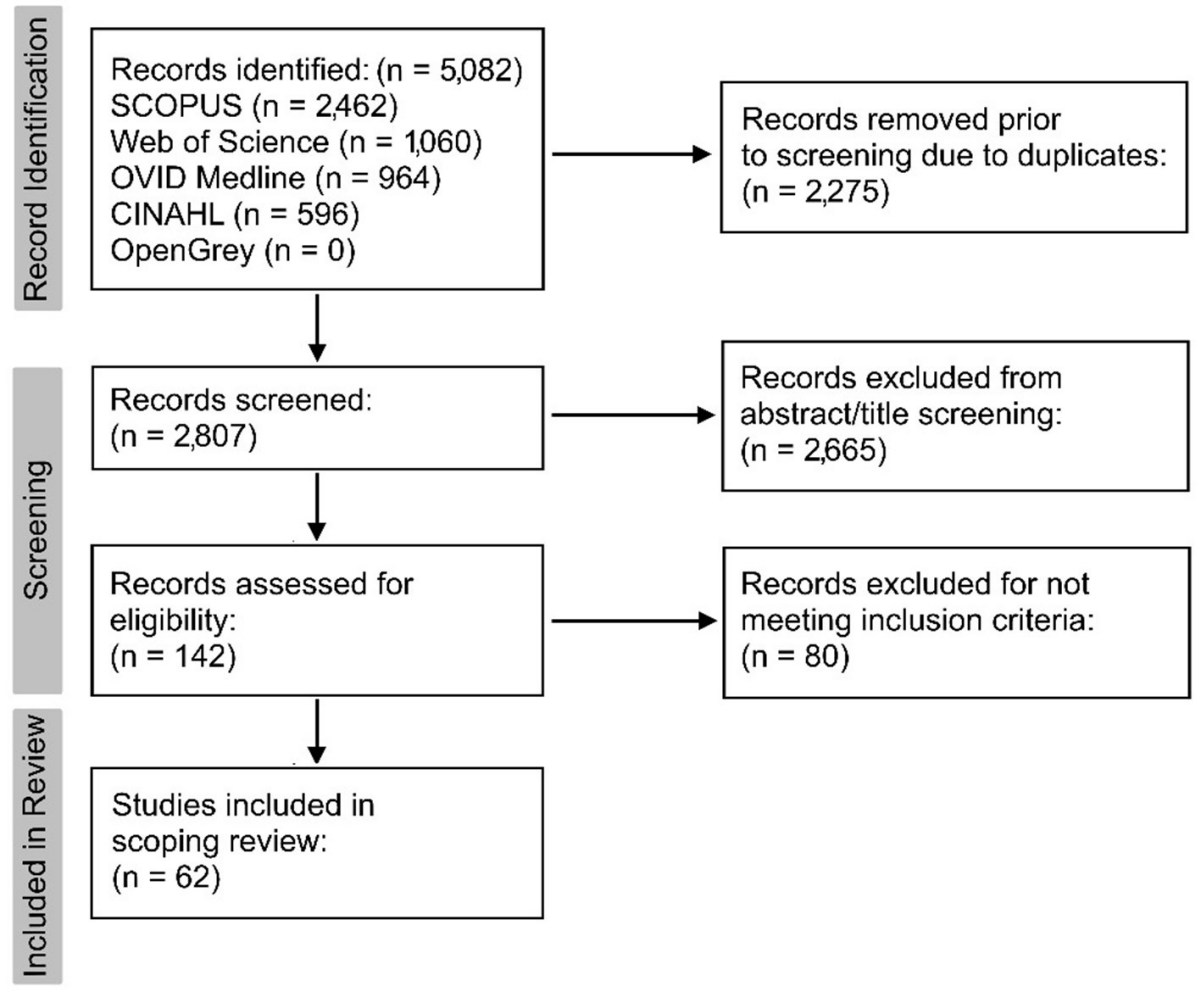
Modified version of the PRISMA 2020 flowchart (9) to summarise record retrieval.

One hundred and forty-two records underwent full-text screening. Of these, 80 did not meet the inclusion criteria for various reasons, including studies that could only be retrieved in abstract form (*n* = 10), records using secondary research (*n* = 17), research exploring well-established diagnostic techniques in a non-novel way (*n* = 35), and studies that were irrelevant to the research question (*n* = 18). Sixty-two relevant records remained for the scoping review and are illustrated in a modified version of the PRISMA flowchart (Figure 1) (9).

Retrieved records were categorised into 5 groups based on their LVO detection method. These included stroke scales (*n* = 30), imaging and physiological methods (*n* = 15), algorithmic and machine learning approaches (*n* = 9), physical symptoms (*n* = 5), and biomarkers (*n* = 3).

Geographical influence of studies was assessed by grouping records by continent of recruitment. Twenty-six records were based in North America, whilst 17 records were from both Europe and Australasia, respectively. One record was based in South America and one record was from mixed countries.

Most included records featured retrospective, single-centre experiences (*n* = 36), followed by 16 records which reported retrospective, multicentre experiences. Four records used prospective, single-centre recruitment, whilst 3 were prospective, multicentre studies. Finally, 3 records included a mix of retrospective and prospective multicentre experiences.

Fifty-four out of the 62 included articles provided sensitivity and specificity values.

### Stroke Scales

#### Pre-existing Scales

Thirty records investigating stroke scales were included in this review, including 10 records which adapted pre-existing scales for use in LVO (10–19). Stroke scale studies were based on a combination of physical and clinical findings.

The Los Angeles Motor Scale (LAMS) is a popular stroke detection scale, often used in the pre-hospital environment. Three records applied LAMS to a LVO population. Firstly, the standard LAMS scale was validated for LVO and demonstrated good sensitivity (76%) and lower specificity (65%), for a cut-off of ≥4, indicating its use in identifying patients requiring transportation to a CSC (10). Modifications to the LAMS was also used but performed less well, with one record trialling the addition of atrial fibrillation (AF) as a scoring criterion, and another adding speech abnormalities (11, 12).

The Cincinnati Prehospital Stroke Scale (CPSS) was trialled for LVO use, with performance reportedly matching more complex severity scales for a cut-off score of 3 (specificity 88%) (13). The CPSS was commended for its simplicity and ease to teach, allowing rapid LVO detection in the pre-hospital environment and triage to a CSC, although its low sensitivity of 41% and low positive predictive value of 29% limits its use. The CPSS was also investigated by Nehme et al. to determine if a high CPSS score could identify LVO; however, this study did not report sensitivity or specificity values (14).

Another popular LVO stroke scale investigated was the Rapid Arterial oCclusion Evaluation (RACE) scale which was developed in 2013 (15, 16). Approaches were made to simplify the scale, such as omitting certain items from the original RACE scale, including head and gaze deviation and aphasia or agnosia (15), and modifying the scale to create the modified Rapid Arterial oCclusion Evaluation (mRACE) (15). However, performance was worse than the standard RACE scale (20), so modifications of this scale are unlikely to be of benefit.

The remaining records each explored different commonly used scales. Simplification of the existing NIHSS (sNIHSS-EMS) combined severity, LVO prediction, and parallel stroke recognition, resulting in a sensitivity of 70% and a specificity of 81%, for a LVO prediction cut-off score of ≥6 (17). An assessment of various NIHSS subitems and published stroke scores to predict LVO on CT or magnetic resonance angiography found an optimal NIHSS cut-off score of 7 to predict LVO, with 81% sensitivity and 77% specificity (18). Finally, application of the Glasgow Coma scale (GCS) for use in LVO detection was found to have high sensitivity (94%) and specificity (90%), for a cut-off score of <15, and a high negative predictive value (98%) (19).

#### Novel Scales

This scoping review identified 20 novel stroke scores developed since 2015 for LVO detection which had yet to be validated (21–40), each with varying performance (see Supplementary Table S3).

Of the proposed novel scales, 3 scales showed greatest promise. Firstly, the Vision, Aphasia, and Neglect (VAN) score identified LVO patients with 100% sensitivity and 90% specificity, based on patients experiencing weakness, and one of the following symptoms of visual disturbances, aphasia, neglect, and an NIHSS score of ≥6 (21). Next, the Ventura Emergent LVO Score (VES) was tested in a sample of 62 pre-hospital patients, with a sensitivity for LVO of 95% and a specificity of 82% (22). Again, this performance is promising as it exceeds that of the currently used NIHSS score and may support the correct triaging of patients. Finally, the Large ARtery Intracranial Occlusion (LARIO) scale evaluated facial palsy, arm weakness, grip strength, language, and neglect in a simple 5-item scale and was found to have a sensitivity and specificity of 100 and 82%, for a cut-off of >3 (23).

Other scales of note include the Field Assessment Stroke Triage for Emergency Destination (FAST-ED) scale, which has comparable predictive performance to more complex scales, such as the NIHSS, with a sensitivity of 60% and specificity of 89%, for a cut-off score of ≥4 (24). FAST-ED also outcompeted known published scales CPSS (56% sensitivity and 85% specificity for a cut-off of ≥2) and RACE (55% sensitivity and 85% specificity for a cut-off of ≥5). The Prehospital Acute Stroke Severity (PASS) scale should also be noted for its simplicity and 66 and 83% sensitivity and specificity scores across a sample of over 3,000 patients, for a cut-off of ≥2 (25).

LVO may be present in patients with low NIHSS scores (26). The LVO scale developed by do Martins-Filho et al. (26) is a sensitive scale for LVO detection (sensitivity of 85% and specificity of 82%, for a LVO score threshold of ≥63), based on NIHSS at admission and middle cerebral artery vessel attenuation on non-contrast computed tomography (NCCT) (26).

The FAST-PLUS stroke score is another proposed scale which is simple to apply to the clinical environment and displays good sensitivity (93%). However, its low specificity (47%) suggests this is of limited use due to the risk of overwhelming CSCs with false-positives (28).

The Conveniently-Grasped Field Assessment Stroke Triage (CG-FAST) scale could prove to be an effective triaging tool due to its sensitivity of 62% and specificity of 81%, for an optimal cut-off of ≥4 (29). The accuracy of CG-FAST was higher than pre-existing triaging scales, such as FAST-ED and RACE (29).

A further novel scale, the Cincinnati Prehospital Stroke Severity Scale (CPSSS), was designed with the aim of being simple for EMS to perform in-field (31). However, the CPSSS had a sensitivity of 83%, at the expense of a reduced specificity (40%), for a cut-off of ≥2, for AIS patients with LVO (31). Further work is warranted for the CPSSS to determine its clinical value.

New scale Gaze Palsy, Aphasia, Inattention, Arm Paresis, and Atrial Fibrillation (GAI_2_AA) was derived using hemispheric symptoms; when set at an optimal cut-off of ≥3, performance included sensitivity of 88% and specificity of 81% (35). The scale also significantly reduced door-to-puncture time, making it a good in-hospital triaging tool (35). A further scale, the Finnish Prehospital Stroke Scale (FPSS), combined conjugate eye deviation with common stroke signs for identifying the presence of both LVO and stroke (36). The FPSS predicted LVO with a low sensitivity (54%) but with a high specificity (91%) (36).

### Imaging and Physiological Monitoring Methods

Fifteen imaging and physiological monitoring methods were identified, of which 5 were advancements in computed tomography angiography (CTA) (41–45) and 2 used MRI methods (46, 47). The remaining records explored different modalities, including standard CT (48), a triage model combining transcranial ultrasound with clinical assessment (49), cranial accelerometery (50), volumetric impedance phase shift spectroscopy (VIPS) (51), and intraoperative neurological monitoring (IONM) (52). The final 3 studies observed cerebral blood flow velocity (CBFV) waveforms using transcranial Doppler ultrasound (TCD) (53–55).

Whilst CTA is commonly used by some centres, novel applications of this method were trialled with good effect. As a gold standard comparator, sensitivity and specificity values for CTA in acute stroke were previously reported at 96 and 87% (56). For example, Boyd et al. (41) trialled CTA with grayscale inversion for LVO detection distal to M2 and M3 MCA segments, resulting in a sensitivity value of 97% and a specificity value of 97% across different radiologist training levels for LVO detection (41). Thick maximum intensity projection in CTA (a 3D imaging technique for viewing CTA data) was also trialled, with up to 83% sensitivity and 99% specificity for LVO detection (42). Yang et al. (43) further tested CTA using a time-resolved C-arm set-up with a sample of 17 patients with LVO. Between two observers, they reported a sensitivity of 100% and specificity between 94–100%, indicating great advancements on previous work (43). However, the image quality of small cerebral arteries at present may not be sufficient for diagnosis (43).

Alongside novel CTA applications, retrieved records also identified studies altering the way that CTA scans are interpreted. Firstly, Hidlay et al. (44) used a smartphone-based evaluation of scans for detecting LVO, with sensitivity and specificity of 100% across 80 LVO patients, in a retrospective multicentre study. Next, automated attenuation analysis was trialled by Reidler et al. (45) with sensitivity values between 91 and 96% and specificity values between 77 and 83%, for a specificity cut-off of ≥0.70 when applied to their cohort of patients (79 with LVO) (45). The CT-defined hyperdense arterial sign marker for LVO demonstrated reasonably high sensitivity (67%) and specificity (82%) for identifying LVO in ischaemic stroke patients on thin and thick NCCT serial images (48). However, its utility is currently limited as thick and thin CT image slices are not readily available in many hospitals (48).

One imaging modality in particular demonstrated high diagnostic accuracy and reliability (46). 3D black-blood MRI (an imaging technique where the signal from blood flow is suppressed) confidently diagnosed LVO with 100% sensitivity and 100% specificity with both contrast and non-contrast scans, observed with intraluminal T1 hyperintensity and contrast-enhancement imaging criteria (46). A further application of MRI, Fluid Attenuated Inversion Recovery Vascular Hyperintensity, demonstrated excellent diagnostic performance for identifying LVO and good-to-excellent reproducibility, with sensitivity and specificity scores of 98 and 86% (47).

The effectiveness of a triage model combining transcranial ultrasound and clinical assessment was assessed to help select patients for intravenous thrombolysis or MT (49). This approach had excellent specificity (97%) but lacked the ability to identify 55% of patients with potential LVOs (49).

Cranial accelerometery, a headset which measures small head movements created by venous inflow to the brain during cardiac contraction using sensitive accelerometers, was combined with asymmetric arm weakness to assess if diagnostic accuracy was improved compared to each of these detection techniques alone (50). This approach increased sensitivity and specificity scores from 65 and 91% to 91 and 93%, compared to cranial accelerometery alone (50). Another non-invasive LVO detection device, volumetric impedance phase shift spectroscopy (VIPS), is a tool placed on the head like a visor and measures the bioimpedance of each brain hemisphere. VIPS demonstrated excellent sensitivity and specificity (93% and 92% among all stroke patients), suggesting it may be a suitable tool for triage (51).

IONM is a novel parameter currently used for detecting the onset of neurological dysfunction in anaesthetised patients, using modalities such as somatosensory evoked potentials and electroencephalography to precisely define the time of last electrically well (52). The IONM study did not provide sensitivity or specificity values and had a small sample size of 5, so its feasibility is still unclear (52).

Recent extensions to TCD cerebral blood flow monitoring include waveform categorisation by Thorpe et al. (53) who categorised CBFV waveforms into clusters (LVO vs. non-LVO) for assessment using an unsupervised machine learning method. This method may help differentiate between stroke types and could be a useful tool for EMS before a patient arrives at hospital (53). This study did not report sensitivity or specificity values.

A further TCD feasibility study retrospectively compared two metrics for LVO detection: velocity curvature index (VCI), computed from CBFV waveforms, and velocity asymmetry index (VAI), which quantifies differences in CBFV between hemispheres (54). VCI proved superior to VAI and was trialled in a separate further study for detecting LVO, distinguishing between LVO patients and a clinical control group collected in-hospital (55). VCI had an estimated sensitivity of 91% and an 88% specificity for identifying LVO patients for further LVO investigation (55). However, high post-enrolment drop out of subjects in the LVO patient group may have introduced bias into the study.

### Algorithmic and Machine Learning Approaches

Nine studies evaluated the viability of using algorithmic and machine learning methods to predict LVO (57–65). Five of these studies used algorithms to accurately predict LVO from imaging methods, such as CT images (57, 60, 62–64). One study established an automated evaluation system which contained three hierarchical models based on patients' demographic data (model 1), clinical data (model 2), and NCCT scans obtained from a deep learning model (model 3). This approach had high sensitivity (95%) but much lower specificity (68%) using the eXtreme Gradient Boosting (XGboost) algorithm (57).

A study published by Sugimura et al. (58) created a hierarchical algorithm to detect highly likely and unlikely LVO stroke during emergency helicopter transport to hospital; the algorithm combined several LVO-suggestive clinical factors (including eye deviation, AF, and a systolic blood pressure of ≥180 mmHg) and non-suggestive clinical factors (including no eye deviation or limb paresis) to help appropriately select patients presenting with LVO for transportation to CSCs (58). This study did not specify sensitivity or specificity values, so its accuracy is unknown.

In 2017, Nogueira et al. developed a new smartphone application based on a built-in automated decision-making algorithm and the FAST-ED stroke scale developed in 2016 by Lima et al. (24), to assist EMS in triaging patients in pre-hospital settings (59). Another machine learning algorithm, MeThinksLVO, aimed to identify LVO on NCCT, demonstrating good sensitivity (83%) and specificity (71%). MeThinksLVO was improved following the addition of the NIHSS and time from onset to the model (60).

Chen et al. (61) successfully developed an artificial neural network algorithm to predict LVO in the pre-hospital triage stage using pre-hospital accessible data, including patient demographics, NIHSS scores, and vascular risk factors. This artificial neural network would be an effective pre-hospital tool for identifying LVO as its diagnostic parameters (mean Youden index, sensitivity, specificity, and accuracy) were higher than previously established pre-hospital prediction scales, with sensitivity and specificity values of 81 and 83% (61).

Due to the success of deep learning applications in other brain pathology, a deep convolutional neural network was trialled to assess the feasibility of detecting acute LVOs on multiphase CTA, with sensitivity of 96% and specificity of 81% (62).

To detect anterior circulation LVOs, Amukotuwa et al. (63) retrospectively evaluated the accuracy and speed of a new automated tool, RAPID CTA, by analysing raw CTA patient data for the presence and location of a LVO. To determine the sensitivity and specificity for RAPID CTA, interpretation by experienced neuroradiologists acted as a reference standard (63). RAPID CTA demonstrated high sensitivity (92%) and specificity (81%), making it a suitable tool for formal diagnosis (63). A further study by Amukotuwa et al. (63) evaluated the diagnostic accuracy of an algorithm from experienced neuroradiologists in a multicentre study against a reference standard of reads, achieving excellent diagnostic sensitivity (95%) and good specificity (79%) for LVO. This fast automated detection method draws the radiographer's attention to positive findings on CTA, making this a feasible detection approach for LVO in a clinical setting (64).

The final study in this category was a 3-step triage tool for pre-hospital use to reduce assessment time for patients who do not have LVO, named the Ambulance Clinical Triage for Acute Stroke Treatment (ACT-FAST) (65). This study provided high sensitivity and specificity values following retrospective and prospective validation, with a statistically significant improvement on diagnostic performance of the FAST-ED stroke scale and higher accuracy, specificity, and positive predictive value than current LVO stroke scales (65).

### Physical Symptoms

Out of the 5 studies observing stand-alone physical symptoms included in this review, 2 studies focused on eye deviation (66, 67), 1 study focused on cortical symptoms (68), 1 study focused on complete hemiplegia (69), and 1 study focused on neurological symptoms for predicting LVO (70).

Of the 2 studies focusing on eye deviation (66, 67), 1 study observed for conjugate eye deviation (DeyeCOM sign) on CTA and NCCT (66), which may help to identify stroke patients with cortical deficits. Gaze deviation is identified in the NIHSS score and has shown good specificity in a previous study for AIS (71). The DeyeCOM sign study noted a specificity of 100% and sensitivity of 80% for DeyeCOM(++) patients (patients with conjugate gaze deviation on both NCCT and CTA images) and a specificity of 100% and sensitivity of 86% for DeyeCOM(–) patients (absence of DeyeCOM sign on both scans) (66). These results suggest that sustained DeyeCOM(++) and DeyeCOM(–) are strong predictors of anterior LVO stroke presence and absence. DeyeCOM(–) patients often had lower mean NIHSS scores than LVO patients, suggesting DeyeCOM sign can accurately identify LVO stroke from stroke mimics (66).

Radiological eye deviation predicted LVO in patients with stroke-like symptoms with sensitivity and specificity scores of 71 and 78% (67). However, CTA scans were not performed for every patient in the study's cohort, so patient selection bias cannot be excluded (67). There may have also been other underlying causes of eye deviation, such as seizures, which may have increased the incidence rate of LVO being detected (67).

Cortical symptoms, such as aphasia and neglect, in the absence of motor symptoms, are sensitive indicators for LVO stroke (68). Beume et al. (68) observed cortical symptoms to determine LVO presence. The authors' ratings system was optimised to have a low false positive rate so that patients with low NIHSS scores at initial presentation were not missed (68). Complete hemiplegia demonstrated high specificity for predicting LVO (94%) but lacked sensitivity in both derivation and validation cohorts (69).

Pollard et al. (70) screened for LVOs using a clinical paradigm to classify neurologic symptoms (patients not presenting with focal objective neurologic symptoms would be unlikely to have LVO), achieving high sensitivity (100%) but a low specificity (36%). However, with a positive predictive value of 8% and a low specificity score, this system's strength is limited in clinical settings (70).

### Biomarkers

Three studies demonstrating an association between different biomarkers and LVO presence were identified in this review (72–74). Previous studies have indicated an association between cardiac biomarkers and ischaemic stroke, and AF with LVO, but there is little known about the association of other cardiac biomarkers with LVO (72). One retrospective, single-centre study evaluated the association between biomarkers of cardiac dysfunction (serum troponin levels) and left atrial diameter with LVO on transthoracic echocardiograms in AIS patients, adjusting for risk factors of stroke, AF, and demographic factors (72). An association between serum troponin levels and LVO persisted even after adjustment for AF, but no association was noted between the left atrial diameter and LVO following the addition of AF to a multivariate model (72). This indicates an association between the left atrial diameter and troponin positivity with LVO patients (72). A study published in 2018 by Inoue et al. noted a significant correlation between AF and mean systolic blood pressure >170 mmHg in the presence of LVO (73).

Proteomic profiling of plasma biomarkers exhibited high accuracy for detecting AIS due to LVO and the upregulation of 4 proteins (IGF2, LYVE1, PPBP and THBS1) in LVO patients (74). All 4 biomarkers play a role in blood haemostasis and have excellent predictive values for identifying LVO. This study found elevated levels of IGF2 (an insulin-like growth factor) and LYVE1 (a cell surface receptor on lymphatic endothelial cells), and lower THBS1 levels (a regulator of angiogenesis), to all be independent predictors of a favourable outcome 3 months after suffering from AIS due to LVO (74). Similarly to Inoue et al. (74), higher systolic and diastolic blood pressure were noted in the AIS group compared to a control group.

## Discussion

Data were extracted from 62 studies and categorised into 5 different LVO detection groups (stroke scales, imaging and physiological measurement systems, algorithmic and machine learning approaches, physical symptoms, and biomarkers), highlighting new detection techniques not yet validated or developments in previously known techniques for LVO identification from 2015–2021. This scoping review aimed to review published literature and map recent advancements in LVO detection methods, updating current knowledge in the field.

Improvements in clinical support tools would impact LVO detection and patient triage, providing the opportunity for earlier initial treatment and the potential for reductions in secondary transfers, which has been shown to improve patient outcomes (75). As previously described by Heldner et al. (76), the balance of sensitivity and specificity requirements may change depending on the geographical location of the patient. For example, in a remote pre-hospital environment, a high specificity stroke scale at the expense of a reduced sensitivity may be favoured to reduce unnecessary transfers to CSCs. A high sensitivity stroke scale, at the compromise of a reduced specificity, may be selected in areas close to stroke centres to minimise over triaging (76). Both high sensitivity and specificity are necessary at stroke centres to confirm LVO. Consequently, the environment a patient is in may determine whether sensitivity or specificity is to be favoured, which may influence the LVO detection method selected. It is important to note that this compromise between sensitivity and specificity is just one measure of success for influencing patient outcome and that other measures of success do exist.

### LVO Detection

This scoping review has highlighted several methods for initial LVO detection which could be applicable to a variety of environments, including the pre-hospital environment. These methods primarily include stroke scales, but could include the addition of physical symptoms, physiological measurements, and biomarkers.

Whilst there is an unclear consensus on the optimal stroke scale, scales with high sensitivity could be an ideal initial detection method in patients with suspected LVO. Favouring a higher sensitivity over specificity ensures all patients with LVO are identified for transportation to a stroke facility. Based on the findings of this review, the RACE scale and the GCS could be favourable scales due to their high sensitivity and accuracy for predicting LVO, ease of use, and minimal training required (15, 19). Another scale of note not investigated in this review was the Gaze-Face-Arms-Speech-Time (G-FAST) score which also had a high sensitivity (89%) for identifying LVO, for a cut-off of ≥3 (77). Consequently, G-FAST may help to determine which patients require referral to a CSC for cerebrovascular imaging (77). A study performed by Duvekot et al. (78) compared 8 pre-hospital stroke scales for detecting LVO, noting RACE, G-FAST, and CG-FAST as the best performing scales; all three scales neared similar performance to the NIHSS.

Whilst aiming for high sensitivity for LVO detection is beneficial, there is also a counter argument for an alternative pre-hospital focus of high specificity, particularly in rural areas (76). This would result in fewer false-positive patients being transported to CSCs, helping to reduce the demand on CSCs. As a result, in rural areas, scales such as the novel FPSS could be used for both stroke screening and LVO detection due to its high specificity score of 91% (36), although further EMS validation studies are still required. Schlemm et al. (75) recently calculated multiple centre scenarios for optimal additional delay to IVT for both the “mothership” and “drip and ship” pathways in urban and rural environments, using mathematical modelling. The results of this study suggest that AIS-suspecting LVO patients should be redirected to a CSC if the additional waiting time to IVT is 50 mins in rural environments (75). This differs to urban environments where redirection to a CSC should occur if the additional delay to IVT is <30 mins (75).

In addition to the use of stroke scales, this scoping review has identified that routinely performed physiological measurements may also support detection of LVO. For example, Inoue et al. (73) demonstrated that systolic blood pressure of >170 mmHg and the presence of AF on electrocardiograms could be useful markers for LVO. Ongoing work is required to evaluate the performance of adding these routinely performed physiological measurements to existing stroke scales to validate their addition, but also to ensure investigation does not delay LVO care.

There is also an ever-increasing interest in emerging techniques using non-invasive, portable measurement devices. These devices target a variety of physiological measures, and whilst early in development, could prove critical in enhancing LVO detection. This scoping review highlighted cranial accelerometery which, when combined with the presence of asymmetric arm weakness, was found to outperform numerous LVO prediction scales with a specificity score of 93% (50). In addition to the methods highlighted by this review, the field is also expanding with novel devices investigating the use of ultrasound to detect stroke, with a recent application of transcranial tissue Doppler ultrasound to detect ischaemic stroke (79). As these emerging techniques are further investigated, future work is required to validate them as part of the LVO detection pathway. Whilst they may support LVO detection, it is also important to highlight that confirmatory imaging and these detection methods serve different purposes. Confirmatory imaging is used to provide detailed anatomical images and can also be used to exclude differential diagnoses.

Finally, the simple ambulance triage tool (the ACT-FAST algorithm) shows great potential for use in both urban and rural pre-hospital environments due to its high sensitivity and specificity (65). The algorithm identifies one-sided arm weakness in step 1 and determines which arm is experiencing the weakness in step 2 (65). If a patient has a positive result in step 3 (deficits experienced are not pre-existing, the time of onset is <6 h, the patient was living independently, and the patient is not experiencing a stroke mimic), transfer of the patient to a CSC for LVO investigation is recommended (65). Like the stroke scales, this simplistic detection tool could prove useful in the pre-hospital environment, assisting in LVO detection and early triaging.

### LVO Confirmation

Following admission to a stroke facility, both high sensitivity and specificity are necessary to correctly diagnose LVO. As stated by the AHA Stroke Guidelines (2019), cerebral imaging is recommended for identifying LVO (5). Despite this, high demand and long wait times can place pressure on resources and delay time-to-treatment. As a result, emerging techniques to support diagnosis using confirmatory imaging could prove useful.

To relieve the burden on scan reporting, automated algorithms applied to CTA scans, taken at the time of initial presentation, could be useful in LVO detection due to their high sensitivity scores (62–64, 80, 81). The introduction of machine learning algorithms has been offered as an aid for LVO diagnosis, particularly for less experienced stroke practitioners (81). RAPID CTA is one of the recommended raw data analysis methods due to its high sensitivity and specificity value (63). Alternatives not explored in this scoping review include Viz.ai and Brainomix, which are commonly used systems that interpret stroke imaging using convolutional neural networks (82).

In addition to speeding up image analysis, this scoping review has also identified findings to assist in diagnosis. For example, DeyeCOM sign (eye deviation on CT) has been associated with LVO and could support prompt diagnosis.

Alongside confirmatory imaging, this scoping review has highlighted the potential for proteomic profiling of plasma biomarkers (74). Detecting for upregulation of proteins which play a role in blood haemostasis could be used to indicate LVO presence, allowing for earlier intervention (74). Furthermore, it has also been suggested that elevated levels of IGF2 and LYVE1, and reduced levels of THBS1, could all independently predict a favourable patient outcome at 3 months following LVO (74). Whilst these emerging biomarker techniques have potential clinical applications, their use is less likely of value due to the availability of rapid neuroimaging and lack of rapid testing for biomarkers. Moreover, the use of biomarkers is time-dependant and the time point of measurement, with respect to stroke onset, is of the utmost relevance, due to biomarkers being strongly influenced by the degree and severity of the blood-brain barrier breakdown. The time to reach maximum concentration will vary between biomarkers, and significant latency in stroke onset and changes in serum biomarkers may become troublesome when the stroke onset time is unknown, meaning the maximum concentration of a biomarker may be missed.

### Strengths and Limitations of the Review

A major strength of this review was the prospectively registered comprehensive search of the literature based on a well-developed search strategy. The results of this study are limited by small cohort sizes and retrospective study designs which may introduce selection bias. Some studies only assessed findings from one ethnicity which may not be representative of a more diverse global population. The definition of LVO varies across the literature, so some studies included in this review did not include M2 middle cerebral artery segments in their definition of LVO or exclude posterior circulation findings, such as the posterior cerebral artery, making direct comparison between studies difficult. This review did not include a formal risk of bias assessment which could also be noted as a limitation. Moreover, the reference group of each study is of the utmost importance when reporting values for sensitivity and specificity (all LVO or LVO in the anterior cerebral circulation only). Direct comparison between studies in this scoping review proved difficult due to the different gold standards used for delineating “true LVO”, suggesting heterogeneity between studies. Studies including Purrucker et al. (17) and Antipova et al. (49) referred to the patient's hospital discharge diagnosis as the “gold standard” (17, 49). However, some stroke scale studies, such as Noorian et al. (10), Lawner et al. (16), and Brandler et al. (12), noted the NIHSS as the “gold standard” stroke scale for LVO stroke severity assessment and diagnosis. In addition to this, only 54 out of the 62 studies included sensitivity and specificity values, once again rendering direct comparison between studies more difficult.

### Future Work

Future work in this field should aim to increase cohort sizes, include a representative sample of the whole population, with varying ethnicities, and prospectively validate findings in-field and in clinical settings if possible. Furthermore, heterogeneity between study protocols and populations may be influencing the reported sensitivities and specificities, rendering comparisons between LVO detection methods more difficult. As a result, all future LVO detection studies should aim to compare to a single gold standard of LVO diagnosis, have the same LVO definition, and ensure that sensitivity and specificity values are reported on. Reporting of additional variables in future work could also support comparisons between methods, including all studies reporting the number of occlusions, any previous infarctions or brain scarring, any variations in the vasculature and brain areas supplied in an individual, and any variation in other brain anatomy. All of these variables may alter study outcomes, and therefore clear reporting is required.

## Conclusion

Early recognition and management of LVO can improve patient outcome. This scoping review has explored literature on novel and advancements in pre-existing detection methods for LVO. This review highlights LVO detection methods with good sensitivity and specificity performance, with a discussion regarding favouring sensitivity or specificity depending on the environment a patient is in. Furthermore, this review presents recent advancements used to support current LVO confirmatory imaging, including the use of artificial intelligence and algorithms.

## Author Contributions

JN and JI designed the study, performed screening and data extraction, analysed the results, and contributed to the writing of the manuscript. JM and EC reviewed the manuscript. All authors approved the final version of the manuscript.

## Funding

JN is supported by the Medical Research Council, Grant Number MR/N013913/1. JM is an NIHR Clinical Lecturer in Older People and Complex Health Needs.

## Author Disclaimer

The views expressed in this publication are those of the author(s) and not necessarily those of the NHS, the National Institute for Health Research, or the Department of Health, or the authors' respective organisations.

## Conflict of Interest

The authors declare that the research was conducted in the absence of any commercial or financial relationships that could be construed as a potential conflict of interest.

## Publisher's Note

All claims expressed in this article are solely those of the authors and do not necessarily represent those of their affiliated organizations, or those of the publisher, the editors and the reviewers. Any product that may be evaluated in this article, or claim that may be made by its manufacturer, is not guaranteed or endorsed by the publisher.
