# Supplementary material for: Emerging Detection Techniques for Large Vessel Occlusion Stroke: A Scoping Review
Source: Front Neurol. 2022 Jan 6;12:780324. doi: 10.3389/fneur.2021.780324 (PMC8796731; doi:10.3389/fneur.2021.780324)
Supplement: Supplementary file 1 [file Table_1.DOCX]

**Emerging Detection Techniques for Large Vessel Occlusion Stroke: A Scoping Review.**

**Supplementary**

**Table S1.** The search strategy used for the scoping review.

|  | Search strategy |
| --- | --- |
| LVO terms | “large vessel occlusion” OR “LVO” OR “large vessel occlusion stroke” OR “LVO stroke” |
| Methods of LVO detection | “ultrasonography, Doppler” OR “ultrasonography” OR “ultrasonography, Doppler, transcranial” OR “transcranial Doppler ultra*” OR “TCD” OR “brain imaging” OR “neuroimaging” OR “near infrared spectroscopy” OR “spectroscopy, near-infrared” OR “NIRS” OR “near-infrared spectroscopy” OR “tomography, X-ray computed” OR “imaging CT” OR “computerised tomography” OR “magnetic resonance imaging” OR “MRI” OR “TCCD” OR “ultrasonography, Doppler, colour” OR “transcranial tissue Doppler” OR “microwave technology” OR “stroke assessment scale*” OR “non-contrast head CT” OR “noncontrast head CT” OR “NCCT” OR “stroke MRI” OR “CT scan” OR “CT angiography” OR “CTA” OR “computed tomography angiography” OR “CT-angiography” OR “magnetic resonance angiography” OR “vascular imaging” OR “Doppler ultrasound” OR “ultrasound” OR “ultrasonography, Doppler, duplex” OR “nuclear medicine” OR “tomography, emission-computed” OR “positron-emission tomography” OR “diagnostic imaging” OR “tomography, emission-computed, single-photon” OR “nuclear medicine imaging” OR “PET scan” OR “SPECT scan” OR “CT perfusion” OR “MR perfusion” OR “MR venography” OR “magnetic resonance venography” OR “microwave imaging technique*” OR “assessment” OR “screen*” OR “TCTD” OR “clinical stroke sc*” OR “stroke sc*” OR “pre-hospital screening tool” OR “prehospital screening tool” OR “pre-hospital stroke sc*” OR “prehospital stroke sc*” OR “artificial intelligence” OR “AI” OR “national institute of health stroke scale” OR “NIHSS” OR “rapid arterial occlusion evaluation” OR “RACE” OR “field assessment stroke triage for emergency destination” OR “FAST-ED” OR “Cincinnati prehospital stroke severity scale” OR “Cincinnati pre-hospital stroke severity scale” OR “CPSSS” OR “3-item stroke scale” OR “3I-SS” OR “prehospital acute stroke severity scale” OR “pre-hospital acute stroke severity scale” OR “PASS” or “screening tool” OR “algorithm” OR “pre-hospital sc*” OR “prehospital sc*” OR “multiphase CT angiography” OR “multiphase CTA” OR “machine learning” OR “random forest learning” OR “RFL” OR “convolutional neural network” OR “CNN” OR “Cincinnati pre-hospital stroke scale” OR “Cincinnati prehospital stroke scale” OR “CSTAT” OR “face arms speech test” OR “FAST” OR “Los Angeles pre-hospital stroke screen” OR “Los Angeles prehospital stroke screen” OR “LAPSS” OR “Los Angeles motor scale” OR “LAMS” OR “Miami emergency neurological deficit” OR “MEND” OR “ABCD” OR “ABCD2” OR “Scandinavian stroke scale” OR “SSS” OR “Canadian neurological stroke scale” OR “CNSS” OR “gaze face arms speech time” OR “G-FAST” OR “Cincinnati stroke triage assessment tool” OR “C-STAT” OR “ASTRAL-occlusion score” OR “acute stroke registry and analysis of Lausanne-occlusion score” OR “Japan urgent stroke triage scale” OR “JUST” OR “field assessment of critical stroke by emergency by emergency services for acute delivery to a comprehensive stroke center” OR “field assessment of critical stroke by emergency by emergency services for acute delivery to a comprehensive stroke centre” OR “FACE2AD” OR “gaze palsy, aphasia, inattention, arm paresis, atrial fibrillation” OR “GAI2AA” OR “ACT-FAST” OR “ambulance clinical triage for acute stroke treatment” OR “stroke network of Wisconsin” OR “SNOW” OR “AlphaStroke” OR “serum biomarkers” OR “cardiac biomarkers” OR “Minnesota prehospital stroke scale” or “MPSS” or “Ventura emergent large vessel occlusion score” OR “FAST PLUS” OR “face arm speech test plus severe arm or leg motor deficit” OR “predictive sc*” OR “conveniently grasped field assessment stroke triage” OR “CG-FAST” OR “emergency medicine stroke assessment” OR “EMSA” OR “emergent large vessel occlusion” OR ELVO” OR “Finnish prehospital stroke scale” OR “FPSS” OR “lower extremity strength, eyes/visual fields, gaze deviation, speech difficulty” OR “LEGS” OR speech, arm, vision, eyes” OR “SAVE” OR “prehospital acute stroke triage” OR “pre-hospital acute stroke triage” OR “PAST” OR “recognition of stroke in the emergency room” OR “ROSIER” |

**Table S2.** The data charting form used for the scoping review to extract the relevant information.

| **Data Items** | **Associated questions** |
| --- | --- |
| **General article information** | |
| Title | What was the title of the study? |
| Authors/affiliations | Who was the study carried out by? |
| Type of literature published | What type of literature is the source? Journal, grey literature, conference proceeding etc. |
| Year published | What year was the study published? |
| Geographical location of study | Where did the study occur? |
| Name of method of detection | What is the method of detection called? |
| **Study** | |
| Study design | What was the design of the study? What stage is the method of detection currently being tested at? Is the method of detection already known and being developed further? |
| Study aims | What were the aims of the study? |
| Population | What population was studied (age/gender) and were there any inclusion/exclusion criteria for the study? |
| Study size | What was the sample size? |
| Method of detection approach | How does the method of detection work? What is the approach for LVO detection? |
| Sensitivity of this detection method | What is the sensitivity of this method of detection for LVO? |
| Specificity of this detection method | What is the specificity of this method of detection for LVO? |
| Advantages of this detection method | What advantages does this method of detection offer? |
| Limitations of this detection method | What are the limitations of this method of detection? |

**Table S3.** Summary of the data extracted for novel stroke scales included in the scoping review.

| **New Stroke Scales** | **Author** | **Year published** | **Study design** | **Sample size** | **Sensitivity for LVO** | **Specificity for LVO** |
| --- | --- | --- | --- | --- | --- | --- |
| Stroke Vision, Aphasia, Neglect (VAN) (21) | Teleb et al. | 2017 | Prospective, single-centre study | 62 (14 with LVO) | 100% for VAN positive patients | 90% for VAN positive patients |
| Ventura Emergent Large Vessel Occlusion Score (VES) (22) | Taqi et al. | 2019 | Retrospective, single-centre study | 194 (38 with LVO) | 95% | 87% |
| Large ARtery Intracranial Occlusion Stroke Scale (LARIO) (23) | Vidale et al. | 2019 | Prospective, single-centre study | 145 (54 with LVO) | 100% for a cut-off of >3 | 82% for a cut-off of >3 |
| The Field Assessment Stroke Triage for Emergency Destination (FAST-ED) (24) | Lima et al. | 2016 | Retrospective, multi-centre study | 741 (240 with LVO) | Best performance = 71% for a cut-off of ≥3 and 60% for a cut-off of ≥4 | Best performance = 78% for a cut-off of ≥3 and 89% for a cut-off of ≥4 |
| Prehospital Acute Stroke Severity Scale (PASS) (25) | Hastrup et al. | 2016 | Retrospective, multi-centre study | 3,127 (1,104) | 66% for a cut-off of ≥2 | 83% for a cut-off of ≥2 |
| LVO score derived from the NIHSS and NCCT (26) | Kleber do V. Martins Filho et al. | 2019 | Retrospective, single-centre study | 178 (58 with LVO) | 85% for a LVO score threshold of ≥63 | 82% for a LVO score threshold of ≥63 |
| Field Assessment of Critical Stroke by Emergency Services for Acute Delivery (FACE2AD) (27) | Okuno et al. | 2020 | Both, multi-centre study | 787 (86 with LVO) | 80% | 78% |
| FAST-PLUS Test (28) | Václavík et al. | 2018 | Prospective, multi-centre study | 435 (124 with LVO) | 93% | 47% |
| Conveniently-Griped Field-Assessment Stroke Triage (CG-FAST) (29) | Gong et al. | 2019 | Retrospective, single-centre study | 1,355 (664 with LVO) | 62% for a cut-off of ≥4 | 81% for a cut-off of ≥4 |
| Emergency Medical Stroke Assessment (EMSA) (30) | Gropen et al. | 2018 | Retrospective, single-centre study | 1,663 (171 with LVO) | 75% for a cut-off of ≥3 | 50% for a cut-off of ≥3 |
| Cincinnati Prehospital Stroke Severity Scale (CPSSS) (31) | Katz et al. | 2015 | Retrospective, multi-centre study | 650 (222 with LVO) | 83% for a cut-off of ≥2 | 40% for a cut-off of ≥2 |
| Austrian Prehospital Stroke Scale (APSS) (32) | Krebs et al. | 2021 | Both, single-centre study | 741  (323 with LVO) | 69% | 85% |
| Clinical Information, Vital Signs, and Initial Labs (CIVIL) (33) | Lee et al. | 2020 | Retrospective, single-centre study | 1,621 (291 with LVO) | 67% for a cut-off of ≥3 | 80% for a cut-off of ≥3 |
| Prehospital acute stroke triage (PAST) (34) | Li et al. | 2020 | Retrospective, single-centre study | 2,499 (311 with LVO) | 85% for a cut-off of ≥2 | 76% for a cut-off of ≥2 |
| Gaze Palsy, Aphasia, Inattention, Arm Paresis, and Atrial Fibrillation (GAI_2_AA) (35) | Ohta et al. | 2019 | Both, multi-centre study | 1,130 (144 with LVO) | 88% for a cut-off of ≥2 | 81% for a cut-off of ≥2 |
| The Finnish Prehospital Stroke Scale (FPSS) (36) | Ollikainen et al. | 2018 | Retrospective, multi-centre study | 856 (107 with LVO) | 54% | 91% |
| Pomona Scale (37) | Panichpisal et al. | 2018 | Retrospective, single-centre study | 776 (94 with LVO) | 86% for a cut-off of ≥2 | 70% for a cut-off of ≥2 |
| Emergent LVO Screen (38) | Suzuki et al. | 2018 | Prospective, multi-centre study | 413 (114 with LVO) | 85% for a cut-off of ≥1 | 72% for a cut-off of ≥1 |
| Japan Urgent Stroke Triage (JUST) (39) | Uchida et al. | 2018 | Prospective, multi-centre study | 1,007 (131 with LVO) | 84% for a cut-off level of 4 | 69% for a cut-off level of 4 |
| Five-Item Score (40) | Vanacker et al. | 2016 | Retrospective, multi-centre study | 2,023 (566 with LVO) | 84% for a cut-off of 16 | 71% for a cut-off of 16 |
